# Monitoring dynamic cytotoxic chemotherapy response in castration-resistant prostate cancer using plasma cell-free DNA (cfDNA)

**DOI:** 10.1186/s13104-019-4312-2

**Published:** 2019-05-15

**Authors:** Katherin Patsch, Naim Matasci, Anjana Soundararajan, Patricia Diaz, David B. Agus, Daniel Ruderman, Mitchell E. Gross

**Affiliations:** Lawrence J. Ellison Institute for Transformative Medicine, University of Southern California, Los Angeles, CA USA

**Keywords:** Cell-free DNA, Chemotherapy, Prostate cancer, Androgen receptor, Sequencing

## Abstract

**Objective:**

Cell-free DNA (cfDNA) is an attractive cancer biomarker, as it is thought to reflect a component of the underlying genetic makeup of the tumor and is readily accessible in serial fashion. Because chemotherapy regimens are expected to act rapidly on cancer and cfDNA is cleared from the blood within minutes, we hypothesized that cfDNA would reflect immediate effects of treatment. Here, we developed a method for monitoring long cfDNA fragments, and report dynamic changes in response to cytotoxic chemotherapy.

**Results:**

Peripheral blood was obtained from 15 patients with metastatic castration-resistant prostate cancer (CRPC) immediately before and after cytotoxic chemotherapy infusion. cfDNA was extracted and quantified for long interspersed nuclear elements (LINE1; 297 bp) using qPCR. Targeted deep sequencing was performed to quantify the frequency of mutations in exon 8 of the androgen receptor (AR), a mutational hotspot region in CRPC. Single nucleotide mutations in AR exon 8 were found in 6 subjects (6/15 = 40%). Analytical variability was minimized by pooling independent PCR reactions for each library. In 5 patients, tumor-derived long cfDNA levels were found to change immediately after infusion. Detailed analysis of one subject suggests that cytotoxic chemotherapy can produce rapidly observable effects on cfDNA.

**Electronic supplementary material:**

The online version of this article (10.1186/s13104-019-4312-2) contains supplementary material, which is available to authorized users.

## Introduction

Recent studies have begun to elucidate the relationship between molecular marker status in the blood and therapy response in castration-resistant prostate cancer (CRPC) [1–3]. At the genetic level, AR point mutations are associated with resistance to therapies [1, 2, 4–7]. Clinically, response to therapy is typically evaluated via measurement of prostate-specific antigen (PSA) at regular intervals, with PSA decline rate being shown to inform on overall survival [8, 9]. However, PSA response can take weeks to months to present, and changes may be hard to interpret, as they can reflect both PSA release from cell lysis and production from intact cells. Hence, to develop personalized treatment strategies there is a need to more rapidly monitor patients while receiving cancer treatment and to obtain information reflective of the molecular make-up of their tumors.

Plasma cell-free DNA (cfDNA) can be readily accessed in serial fashion to analyze the dynamic clonal make-up of cancers across tumor evolution and therapy [1, 10–22]. This is especially valuable in metastatic disease, where sequential access to tissues is not feasible. In prostate cancer, researchers have used cfDNA to track changes in AR mutation status in response to the targeted therapies abiraterone and enzalutamide [1, 2, 19, 20, 23, 24]. Both overall cfDNA levels and specific mutations have been suggested to inform on response to therapy and outcome [21, 22, 25–27].

Previous studies analyzed cfDNA after several weeks to months of treatment. The goal of the current study was to look for therapy effects at shorter time frames, just hours after administration of therapy. Because the half-life of cfDNA is < 1 h [28–31], this approach may offer the potential for monitoring dynamic changes in response to treatment. We therefore developed a targeted approach to track AR mutations in plasma to attempt to correlate with systemic response in CRPC patients at the time of chemotherapy administration.

## Main text

### Materials and methods

#### Patient cohort

Plasma was obtained from patients with metastatic prostate cancer treated with docetaxel-based chemotherapy as part of a correlative clinical trial. Dexamethasone and antiemetics were administered as per standard of care. Serial samples were obtained immediately before, 30–60 min after, and in our case study, 24 h after docetaxel infusion. Baseline patient demographics are presented in Additional file 1.

#### Sample processing

EDTA tubes of whole blood were collected and processed within 2 h. Plasma was removed after centrifugation at 1600 rpm at 4 °C and stored in 1 ml aliquots at − 80 °C.

#### cfDNA extraction and quantitation

Plasma samples were thawed on ice and centrifuged at 4 °C to remove residual cells. cfDNA was extracted from 200 µl aliquots using the QIAamp DNA Mini Blood Mini Kit (Qiagen). Samples were eluted in 100 µl and 2 µl were quantified for long interspersed nuclear elements (LINE1, 297 bp amplicon) using qPCR as described previously [32].

#### Amplification of the androgen receptor exon 8

Two sequences that spanned exon 8 of the AR were amplified using Phusion polymerase (New England Biolabs) and the following primers: 5′-CCACCTCCTTGTCAACCCTG-3′ and 5′-GATCTCTGCCATCATTTCCGG-3′ for amplicon A1 (138 bp), and 5′-CAAGTCACACATGGTGAGCG-3′ and 5′-GGTTTCCAATGCTTCACTGGGT-3′ for amplicon A2 (128 bp). DNA (1 µl) was amplified as follows: (1) 98 °C 30 s, (2) 30 cycles: 98 °C 10 s, 58 °C 10 s, 72 °C 10 s, (3) 72 °C 5 min. PCR products were purified using Agencourt AMPure XP beads at 1.2× concentration (Beckman Coulter). Reactions were eluted in 10 µl.

#### Targeted sequencing of androgen receptor exon 8

Amplicon DNA concentrations were determined using the BioAnalyzer with the High Sensitivity DNA Analysis Kit (Agilent Technologies). Libraries were generated with Kapa Hyper Prep Kit (Kapa Biosystems). Barcoded libraries were quantified using the BioAnalyzer, pooled and sequenced using an Illumina MiSeq. For each sample, a pool of 10 independent PCR reactions was analyzed to reduce technical variability. Results from a single patient sample with two single nucleotide variants (SNVs) are demonstrated in Additional file 1, where results from the pooled sample were equivalent to the average of the observed frequencies of each product weighted by its coverage.

To estimate the sensitivity of our approach, samples with known SNV frequencies were sequenced. We amplified variants of the AR using two different plasmids as template, (1) pEGFP-C1-AR [33] (a gift from Dr. Michael Mancini, Addgene plasmid # 28,235) (AR-LBD WT) and (2) p270 pCMV-CRE-M-AR(LNCaP) (a gift from Dr. Jeffrey Green Addgene plasmid # 12,496) (AR-LBD T878A). Amplicons were generated using the following primers: 5′-CCTGCTCAAGACGCTTCTACC-3′ and 5′-TCACTGGGTGTGGAAATAGATGG-3′. Purified amplicons were diluted to 350 ng/ml final concentration, mixed at various ratios and sequenced. Additional file 2 compares the observed fraction (99.6%, 78.0%, 50.4%,13.3%, 5.9%, 0.4%) with the actual fraction of mutant DNA (100%, 75%, 50%, 10%, 5%, 0%), highlighting the high accuracy of our pipeline in quantifying SNVs (R^2^ = 0.999) with low absolute error (root mean square RMS = 1.9%).

#### Data analysis

After demultiplexing and adapter/barcode trimming, reads were mapped to human chromosome X (hg19) using *BWA* v. 0.7.9 [34]. FixMateInformation and MarkDuplicateWithMateCigar from the package *picard* v. 1.130 (http://broadinstitute.github.io/picard) were used to ensure and verify correct mate-pair information. Resulting alignments were sorted and indexed using *samtools* v. 1.3.1 [35] and visually inspected using the *Integrated Genome Viewer* [36]. Variant positions in the target region were identified via visual inspection and read counts for all observed nucleotides at the variant position were obtained using custom Java code based on the *picard* library (http://broadinstitute.github.io/picard). The variant frequency was calculated as the number of reads with the observed alternate allele divided by the total number of reads at that position and expressed as a percentage. Statistical analyses were performed using the statistical language *R* v. 3.2.3 (http://www.R-project.org/) and *RStudio* v. 0.99 (http://www.rstudio.com/).

### Results

#### Patient samples

cfDNA was isolated from 43 plasma samples from 15 subjects and analyzed with qPCR of LINE1 (297 bp), a method in previous studies used to quantify yields (Additional file 1) [32, 37]. The median (range) LINE1 value observed across all samples was 13.1 (0.4–819.8) ng/ml. In the 17 sample pairs obtained immediately before and after docetaxel administration, LINE1 appeared to decrease after chemotherapy (pre: 13.1 (1.3–143.6) vs. post: 8.5 (0.4–311.1 ng/ml). However, a Wilcoxon signed rank test showed that the change was not significant (V= 69, p = 0.75).

#### Targeted sequencing of AR exon 8

We amplified two regions spanning exon 8 of the AR, which contains a cluster of AR mutation hotspots reported in CRPC [38]. Amplified product was used for library construction and subsequent massive parallel sequencing (median depth > 1.6 million reads). We processed a total of 34 samples from 15 patients with a mean Q30 of 95.3%, SD 1.9% (Additional file 1), and clinically informative genomic profiling of cfDNA was feasible in all samples.

#### Detection of genomic alterations in AR exon 8 in patients with CRPC

To identify patients with mutations in the exon 8 of AR, we sequenced cfDNA from 15 patients’ plasma at a single time point. We set our threshold of SNV identification to ≥ 1% frequency. The cut-off was chosen based on our estimate of approximately 100 AR template molecules in each PCR reaction (rough calculation based on LINE1 DNA concentration). Assuming cfDNA is for the most part derived from tumor, we considered 1% a conservative threshold and appropriate for these analyses. Exact frequencies are reported in Additional file 1. Using this threshold, 6 (40%) of the 15 patients analyzed were found to have at least one SNV (Additional file 1). These results are in line with previously reported data [4, 5]. A total of 5 SNVs were identified across all time points, 4 of which were previously reported in CRPC: H875Y and T878A, D891H and Q903H (Table 1, Additional file 1). The exception D880Y has been associated with androgen insensitivity syndrome [39], but not with cancer. Genomic coordinates and COSMIC database IDs are listed in Additional file 1.

**Table 1:** SNVs identified in AR exon 8 in patients with CRPC

Patient	% H875Y	% T878A	% D880Y	% D891H	% Q903H
P3		0–1	0–1		
P7		0–1		4–84	
P10	1				
P11		0–2			
P12		2–6			0–2
P17	3–15	7–31			

List of patients with at least one AR mutation. Specific SNVs listed in subsequent columns. Numbers indicate the frequency range of SNVs measured across sequential samples for each patient. The detection threshold for valid SNVs was set to frequencies ≥ 1%.

#### Rapid changes in cfDNA post cytotoxic chemotherapy

**Fig. 1:**
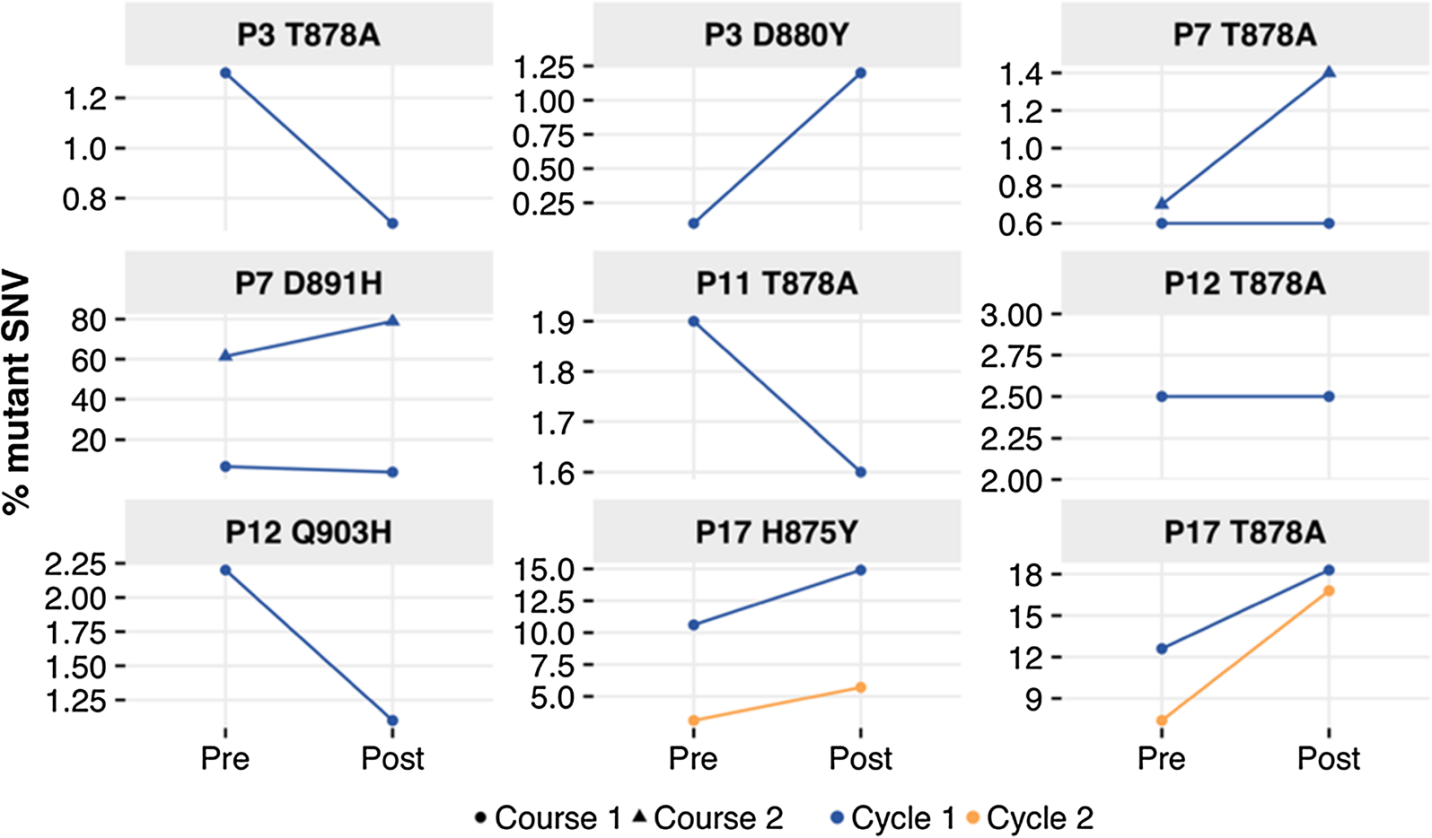
Rapid changes in SNV frequency of cfDNA in response to docetaxel infusion. Mutation frequencies measured in patients’ pre- and post-samples

Next, we asked whether the taxane-based chemotherapy could affect the dynamics of tumor-derived cfDNA immediately after administration. Therefore, we expanded our sequencing assay to sequential sample draws from patients with detected AR mutations. In 5 patients, we detected SNVs at ≥ 1% frequency in both pre- and post-chemotherapy samples (Fig. 1, Additional file 1). In patient P7, we measured the greatest change in SNV frequency after chemotherapy (61% to 79%). Due to considerable changes in two SNV frequencies in Patient 17, we decided to analyze the case in more detail.

#### Case study

Patient P17 had metastatic prostate cancer initially treated with androgen deprivation therapy (leuprolide acetate and bicalutamide) for 4 months before progression to castration-resistant disease. He was then enrolled in a clinical trial and treated with 4 cycles docetaxel with bevacizumab and everolimus [40] and responded with a transient 49% decline in PSA followed by rapid clinical progression with PSA elevation and symptomatic bone pain. The patient died from prostate cancer 5 months after docetaxel was initiated.

**Fig. 2:**
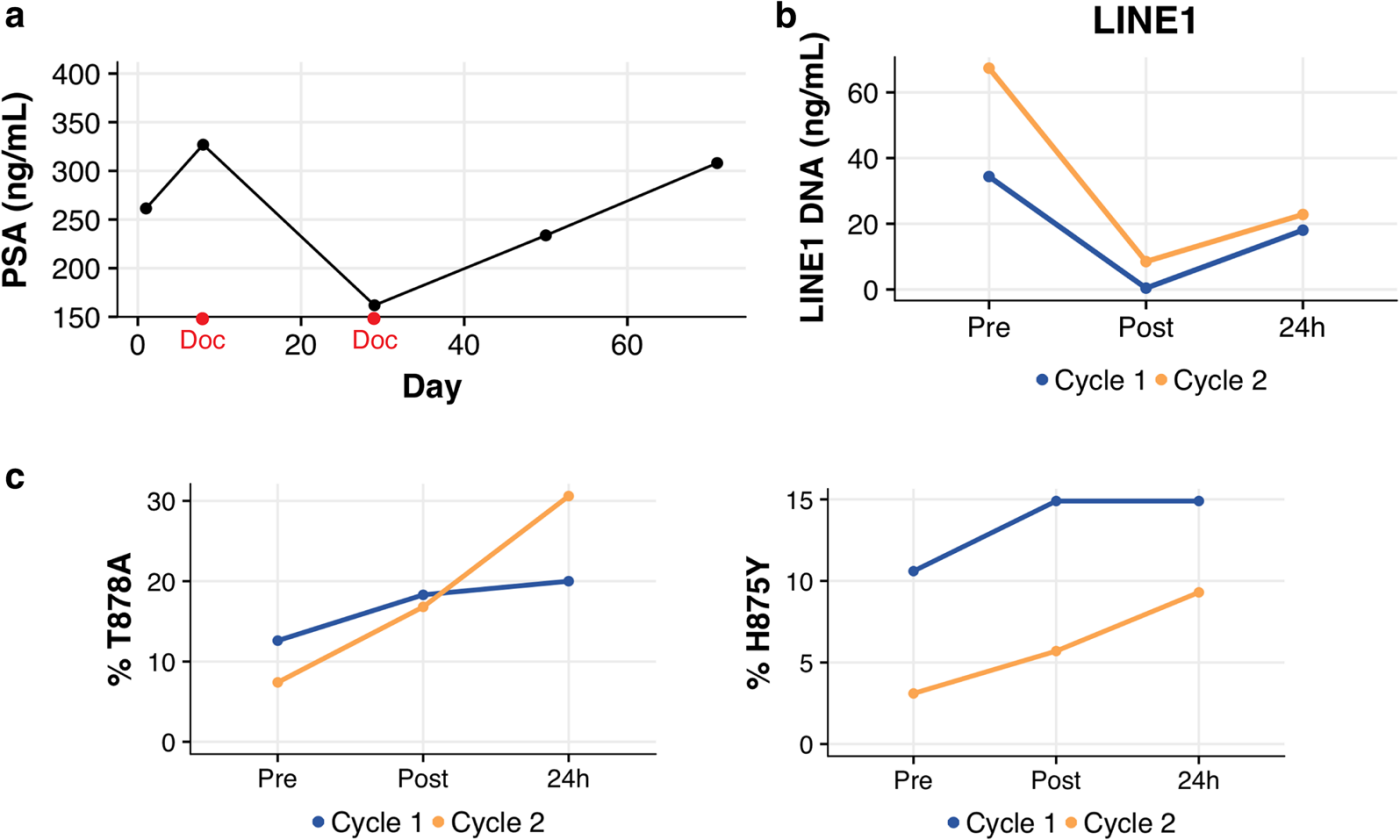
Dynamic response of cfDNA in patient P17 in two consecutive cycles of chemotherapy. cfDNA was isolated from plasma samples collected pre, post and 24 h after infusion of docetaxel at cycles 1 and 2, 21 days apart. **a** PSA plotted over time. Doc markings indicate treatment start dates. **b** LINE1 qPCR to quantify cfDNA dynamics. **c** Mutation frequencies of T878A and H875Y AR

Figure 2 depicts the patient’s dynamic response across treatment. Prior to the first cycle of treatment, total cfDNA content measured by LINE1 was 34.4 ng/ml at baseline. Targeted deep sequencing of AR exon 8 revealed two independent mutations at 11% (H875Y) and 13% (T878A), respectively. Since the sequencing reads contained one or the other alteration, and AR amplifications and mutations are described as mutually exclusive [19, 41], we conclude that the 2 mutations represent two different clones within the patient’s tumor. Immediately after docetaxel infusion, LINE1 decreased to 0.4 ng/ml, while the frequencies of the two AR mutations increased to 15% (H875Y) and 18% (T878A). After 24 h, LINE1increased to 18.1 ng/ml, representing a recovery of 52%, while mutant fraction values remained stably elevated at 15% (H875Y) and 20% (T878A) (Fig. 2b, c). The response pattern in cycle 2 was similar: baseline LINE1 was 67.4 ng/ml. The two independent mutations were measured 3% (H875Y) and 7% (T878A). Immediately after docetaxel infusion, LINE1 decreased to 8.5 ng/ml, while the SNV frequencies increased to 6% (H875Y) and 17% (T878A). After 24 h, LINE1 increased to 22.8 ng/ml, representing a recovery of 34%, while mutant fraction values increased further to 9% (H875Y) and 31% (T878A) (Fig. 2b, c).

In summary, our assay measured dynamic changes in long cfDNA fragments (297 bp) within hours of repeated cycles of cytotoxic chemotherapy, suggestive of response. Future studies involving more patients are required to determine the prognostic and theragnostic value of these short-term dynamics.

### Discussion

Recent studies highlight the dynamics of cfDNA to elucidate mechanisms of resistance to targeted therapies in CRPC that are typically acquired over a course of several months [1, 2, 19, 20, 23]. Here, we demonstrate the measurement of changes in cfDNA profiles within 1 h of chemotherapy infusion.

In the subject we present here in detail, we observed a rapid decline in circulating cfDNA content measured by qPCR of long LINE1. The reported mean half-life of DNA entering the circulation ranges from minutes up to a few hours before it is degraded and cleared by the kidneys, liver and spleen [28, 29]. These data support our hypothesis that a rapid response to therapy might be reflected in the blood circulation. To answer this question will require additional studies including subjects without chemotherapy treatment to determine normal fluctuation of cfDNA.

Surprisingly, the decline in long LINE1 in our case study coincided with elevated allele frequency of mutant AR. These results suggest that chemotherapy affected cfDNA derived from the tumor differently than from healthy tissue. Furthermore, the two separate mutations increased at varying rates, indicative of different clonal dynamics. Taken together, these results suggest that the heterogeneous response dynamics of different tumor clones can be closely monitored using cfDNA. If confirmed in a larger patient study, this could impact the development of patient-specific drug combination therapies and dosing strategies through real-time monitoring.

## Limitations

We cannot rule out that differences in concomitant medications or intra-patient variations influenced the results. For example, we did not specifically control for glucocorticoid use although high doses dexamethasone are usually given as pre-medication before docetaxel [42]. Similarly, we did not longitudinally sample patients off of chemotherapy. It is possible that some of the variations we observed reflect underlying hour-by-hour changes independent of therapeutic intervention. Finally, the small number of patients analyzed across various courses of treatment is a limitation.

## Additional files

**Additional file 1.** Patient data summary. Patient demographics in summary and in detail at baseline, PCR technical variability, cfDNA data and COSMIC IDs of SNVs. Abbreviations: PSA, prostate-specific antigen; Hgb, hemoglobin; LINE1, long interspersed nuclear elements 1, Mets = metastases, LN = lymph nodes. Offset (h) indicates sample collection time relative to treatment. G-K represent single PCR reactions, L represents pooled sample containing 10 replicate PCR reactions. Patient samples analyzed in initial screen in bold.

**Additional file 2.** Estimating the accuracy of our deep sequencing pipeline. DNA sequences of WT and mutant AR-LBD (T878A) were PCR amplified and mixed at various ratios to compare observed %T878A with actual (calculated from the mixture) %T878A.

## Data Availability

The datasets used and/or analyzed during the current study are available from the corresponding author on reasonable request.

## Abbreviations

cfDNA: cell-free DNA
CRPC: castration-resistant prostate cancer
LINE1: long interspersed nuclear elements 1
AR: androgen receptor
PSA: prostate-specific antigen PSA
Hgb: hemoglobin
Mets: metastases
LN: lymph nodes
RMS: root mean square
