# Supplementary figures and images for: Monitoring dynamic cytotoxic chemotherapy response in castration-resistant prostate cancer using plasma cell-free DNA (cfDNA)
Source: BMC Res Notes. 2019 May 15;12:275. doi: 10.1186/s13104-019-4312-2 (PMC6521434; doi:10.1186/s13104-019-4312-2)

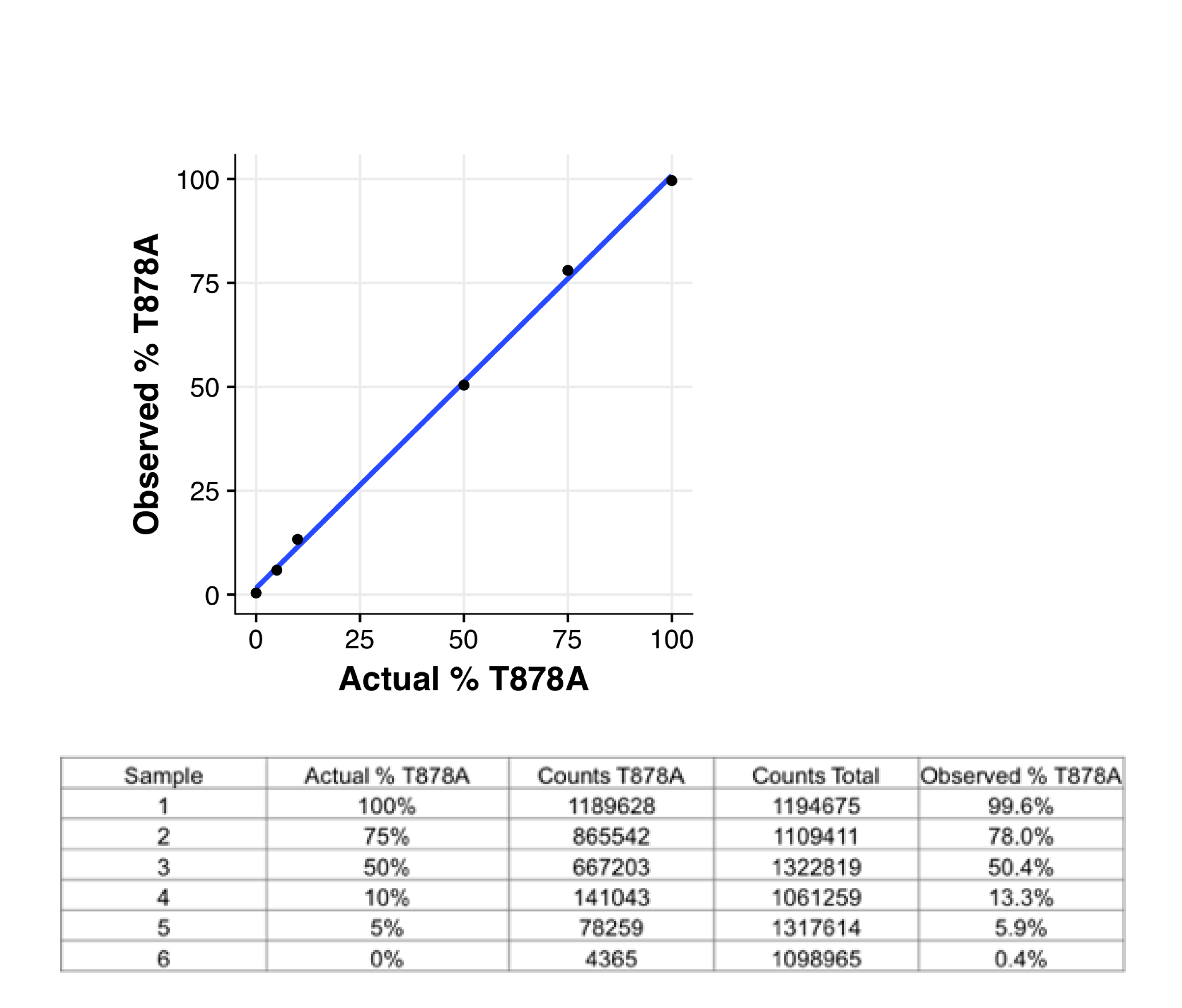

Supplement: Supplementary file 2 — Additional file 2. Estimating the accuracy of our deep sequencing pipeline. DNA sequences of WT and mutant AR-LBD (T878A) were PCR amplified and mixed at various ratios to compare observed %T878A with actual (calculated from the mixture) %T878A. [file 13104_2019_4312_MOESM2_ESM.tif]
